# Pitfalls of Echocardiographic Image Perception: How to Overcome Them?

**DOI:** 10.3389/fmed.2022.850555

**Published:** 2022-04-15

**Authors:** Marina Leitman, Vladimir Tyomkin, Ronen Beeri

**Affiliations:** ^1^Department of Cardiology, Yitzhak Shamir Medical Center, Sackler School of Medicine Tel-Aviv University, Tel Aviv, Israel; ^2^Department of Cardiology, Yitzhak Shamir Medical Center, Zerifin, Israel; ^3^Heart Institute, Hadassah-Hebrew University Medical Center, Jerusalem, Israel

**Keywords:** digital echocardiography, echocardiography workstations, echocardiography monitors, frame rate, compression

## Abstract

In recent years, the significant development of echocardiography systems has led to a sharp improvement in echocardiographic images' quality. In parallel with this, computerized technologies are also going far forward, which today make it possible to ensure a high level of transmission, storage, and display of echocardiography studies. Despite this, many cardiologists are not familiar with modern computerized technologies' new possibilities and continue to use the old standards. That is why many echocardiography laboratories with the best echocardiography systems work following the old minimalist approach. In this paper, we will look at some of the most common mistakes that result from the improper transmission, storage, and demonstration of echocardiography studies, and describe possible ways to overcome these problems.

## Introduction

The latest recommendations on digital echocardiography were published 15 years ago, in 2005 (1). At those times, echocardiography systems could reproduce a frame rate of 25–30 frames per second, hospital networks could support speeds up to 100 MB/s, and the quality of echocardiography studies wasn't comparable with todays' image quality. JPEG compression came from digital photography and wasn't targeted to the scientific world. Cost-effective JPEG compression became quickly very popular in the world of echocardiography because of its high compression ratio. The latest recommendations on digital echocardiography (1) have reached their main goal: to ensure the transition from video recording to digital images, which, upon receipt, can be transferred and stored in digital format for years and can be extracted from digital hospital archives at any time. With the introduction of the second harmonic and other advanced features, the image quality of echocardiography has rapidly improved. Hospital computer resources have developed significantly and modern hospital networks speed was increased at least 10 times to 1,000 MB/s, and even more. The capacity of the digital archives has significantly increased too, and aggressive JPEG compression is no longer required and even eliminates modern image quality achievements.

In the overwhelming majority of echocardiography laboratories, echocardiography examinations after the acquisition come to the workstations, where they are observed by the physician and then are transferred to the digital archive for long-term storage. For a better understanding of these issues, we arranged a questionnaire in 10 major Israeli hospitals (2). We identified three major problems with echocardiographic image quality: reduced video frame rate, lossy video compression, and workstation monitor refresh rate that does not match the video frame rate. Based on this, we identified three questions for echo labs: the frame rate of source and transmitted video, lossy or lossless compression, and the refresh rate of the workstation monitor. All these data were obtained from the settings of echocardiographic systems and workstations. All three points can lead to misdiagnosis. An additional problem arising from the above is eye strain, which is also discussed here.

### Reduced Frame Rate

**Figure 1:**
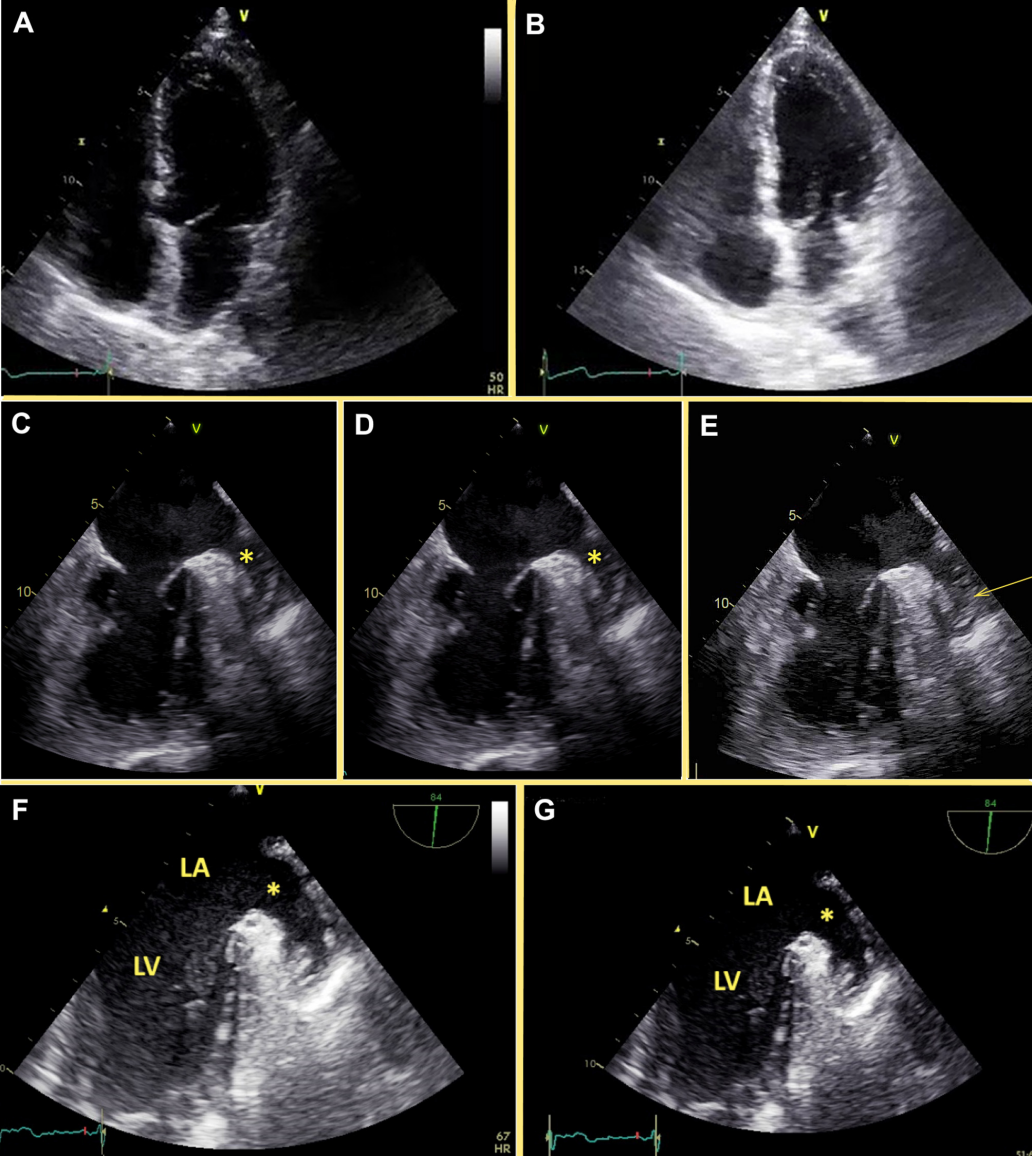
Effect of different frame rates and compression on the echocardiography image quality. **(A,B)** Apical 4-chamber view of the same patients recorded at two different frame rates. **(A)** Left – at frame rate 44 fps, **(B)** Right – at frame rate 74 fps. Image with a higher frame **(B)** rate contains more echocardiography information than the image with a lower frame rate **(A)**. **(C–E)** TEE of the patient with atrial fibrillation before cardioversion. **(C)** Raw data. Swirling is present in the left atrium and the appendage (starr). **(D)** RLE lossless compression. Swirling is present in the left atrium and the appendage (starr). **(E)** Lossy compression. Significant swirling in the left atrium and is suspicious for thrombus in the left atrial appendage (arrow). **(F,G)** TEE of the patient with atrial fibrillation. **(F)** raw data, there is some swirling in the left atrium. **(G)** lossy compression, there is no swirling at all. LV, left ventricle; LA, left atrium.

Today, the typical acquisition frame rate during routine echocardiography examination is 40–90 frames per second (fps), which can reach 120 fps in some cases. It is obvious, that a higher frame rate video contains more image information than video clips with a lower frame rate. This point is particularly important in the strain analysis by the speckle tracking technique. In Figures 1A,B runs from the same patient are reproduced at two different frame rates 44 fps (A) vs. 74 fps (B). The 74 fps image contains much more echocardiography information and has significantly better image quality than the 44 fps image. In many echocardiography laboratories, echocardiography studies are performed using a high acquisition frame rate, but before transmission and storage, the frame rate is reduced. There is no reason to acquire video at a frame rate of 70 fps and observe it at a frame rate of 30 fps on a workstation outside of the echo system. As human vision can differentiate frame rate of 500 Hz (3), the reduction in frame rate is not justified from the echocardiographer's point of view. Indeed, in patients with higher heart rates (e.g., in atrial fibrillation, stress studies, pediatric studies, etc.) significant information seen at acquisition is lost at workstation review after transmission.

### Compression

Compression of echocardiography videos aims to produce smaller digital files for transfer and storage. The maximum compression ratio is achieved using lossy compression (like the JPEG algorithm option). JPEG — Joint Photographic Experts Group, the name of the committee that developed the JPEG standards for lossy image compression algorithms for conventional photography. Echocardiography images are very noisy, so lossy compression may remove useful information and produce artifacts by strengthening noise. As its name implies, lossy compression obtains smaller file sizes at the price of video quality reduction and possible artifacts. In all modern echocardiography systems, there is the option of storing clips using lossless compression without modifying video data (like the RLE algorithm option; RLE - Run-length encoding). Likewise, the widespread availability of cheap storage makes smaller file sizes less material. This compromise in image quality in favor of image compression may result in misdiagnoses when viewing an echo study on a workstation out from the echocardiography machine. One problem that may result from lossy compression is over-diagnosis (Figure 1C), a study acquired without compression demonstrates swirling in the left atrium with no thrombus seen. The same file (Figure 1D), compressed in the lossless RLE compression shows the same finding without loss of information. But, the same file (Figure 1E) reproduced on the workstation in the JPEG lossy format, shows prominent swirling in the left atrium and raises suspicion of a thrombus in the left atrial appendage. This is a clear example of a wrong diagnosis produced by lossy compression with clear clinical implications. The opposite may also happen. Figure 1F shows an original uncompressed still frame of the left atrial appendage, demonstrating some swirling at the left atrium. In Figure 1G, after lossy JPEG compression on the workstation, the swirling is not seen at all.

### Viewing Monitors and Eye Strain

In contrast to radiology, the resolution of echocardiography images is relatively low (1), and monitors with high resolution are not necessary. Conversely, parameters such as refresh rate and response time are crucial in echocardiography monitors. Radiology monitors have high resolution, fixed low refresh rate, and longer response time and thus are not suitable for echocardiography. Regular commercial monitors routinely used in echocardiography have fixed, a non-adaptive refresh rate of 60 or 75 Hz.

**Figure 2:**
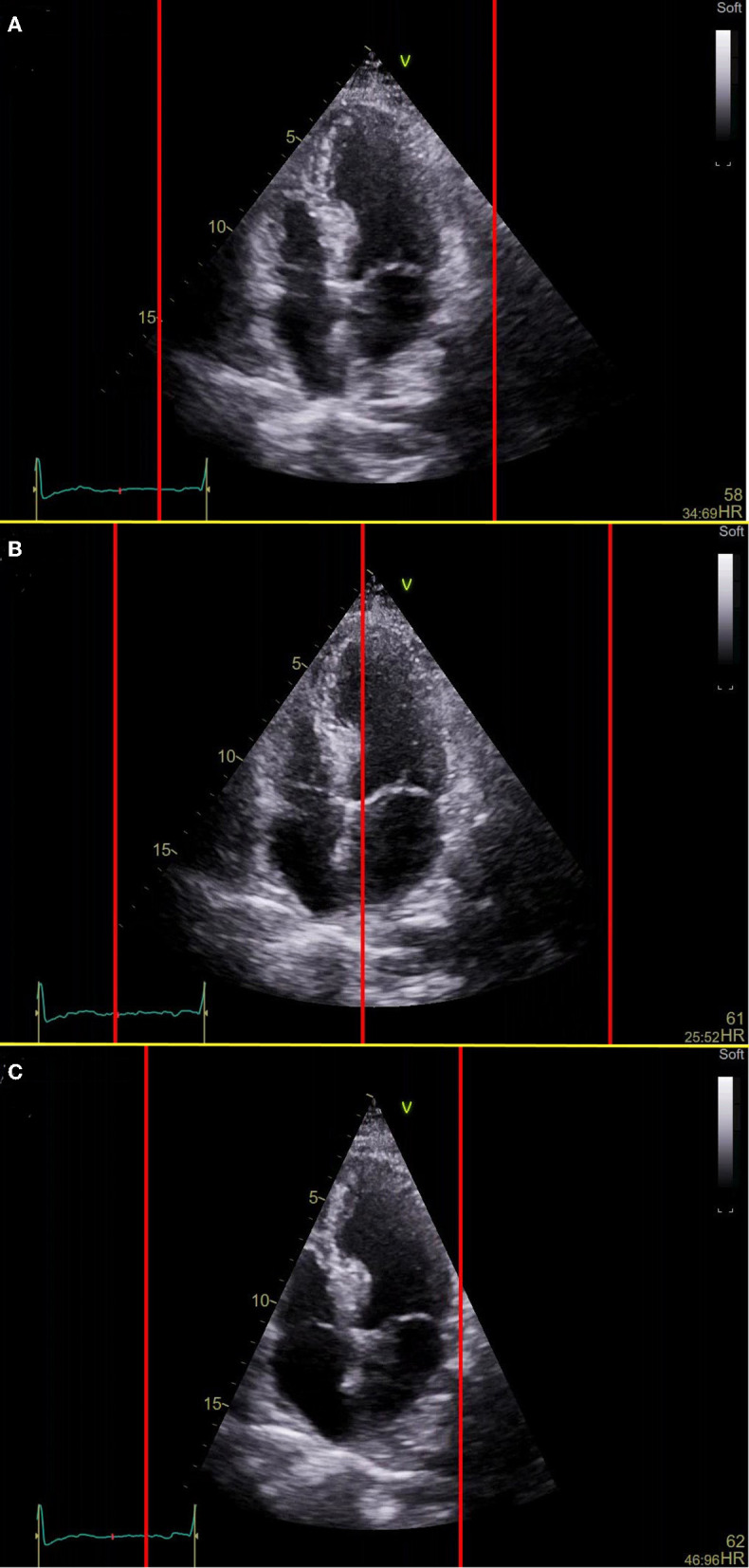
Viewing of the video with different frame rates on the monitor with a fixed refresh rate (60 Hz). **(A)** The frame rate of the video and refresh rate of the monitor is the same. Background video, the apical 4-chamber view has a frame rate of 60 fps, vertical red lines move with a constant speed from frame to frame, in this case, 60 fps. There is an “agreement” between our eyes, red lines, and 4-chamber view. This video is easy for observation and perception. **(B)** The frame rate of the video is lower than the refresh rate of the monitor. Background video, the apical 4-chamber view has a frame rate of 45 fps, and vertical red lines move with a constant speed, simulating the refresh rate of 60 Hz. This video is difficult for perception at 60 Hz monitor, due to the significant jitter effect. **(C)** The frame rate of the video is higher than the refresh rate of the monitor. Background video, the apical 4-chamber view has a frame rate of 90 fps, vertical red lines move with a constant speed (refresh rate of the monitor is 60 Hz). This video is easier for perception than video 3 at 60 Hz monitor, but we also can't observe it for a long time due to the jitter effect and drop of frames.

What does it mean, fixed non-adaptive refresh rate of 60 Hz? This means that the screen is updated (refreshed) 60 times per second. Non-adaptive, fixed refresh rate means that the screen rate does not adapt to the frame rate of the displayed video. Therefore, if a 120 fps video is displayed on a 60 Hz monitor, every 2nd frame is dropped, and the remaining frames are played twice longer. This is known as “drop of frames” (2). In the opposite case, the displayed video obtained at a lower frame rate led to inconsistencies in the sequence of reproduced frames: for example, in a video with a frame rate of 45 fps, 2 frames are played at the rate of 60 fps, and each 3rd frame is twice prolonged at 30 fps (with resulting average frame rate of 45 fps). This problem is known as “the jitter effect” (2). If the video frame rate cannot be divided by the monitor frame rate, some frames are just not rendered and “disappear” (2). This affects significantly the echocardiographer's perception of motion (4). Let's try to reproduce the interaction. In Supplementary Videos 1–3, Figure 2, we will reproduce the interaction between the frame rate of the video, the refresh rate of the monitor, and our perception. In Supplementary Video 1, Figure 2A, the background apical 4-chamber video has a frame rate of 60 fps, and the vertical red lines are moving horizontally with a constant speed. The video will not be distorted on a 60 Hz monitor. In this case, there is an “agreement” between our eyes, red lines, and echocardiography image, 4-chamber view. In Supplementary Video 2, Figure 2B, the background 4 chamber echocardiography view has a frame rate of 45 fps, with red lines moving at a constant speed. We cannot observe this video for a long time on a 60 Hz monitor, due to the significant jitter effect. Red lines move with irregularities, are not in focus. In Supplementary Video 3, Figure 2C, the background 4 chamber view has a frame rate of 90 fps. On a 60 Hz monitor, this is a better option for our eyes than Supplementary Video 2, Figure 2B, but we also cannot concentrate on this video for more than a few seconds, because of irregular motion of red lines, that results from jitter and drop of frames effects. Thus, lack of synchronization between the refresh rate of the monitor and the frame rate of the displayed video affects our perception and causes “motion blur” that affects our eyes, and often is a reason for tired eyes.

## Discussion

In the majority of echocardiography labs, paradoxically, the only person that observes echocardiography examinations in their best original quality is the echocardiography technician, while the physician that is responsible for echocardiography examination deals with compressed images of reduced quality. As we described previously (2), in the vast majority of echo systems, the exported frame rate is set to 30 fps, echocardiography image compression is set to 80-96% of JPEG quality, and monitors of the echocardiography workstations were not suitable for echocardiography examinations (2). All the main compounds that are responsible for echocardiography image quality today can be managed easily, especially in the era of digital archives, which might be easily accessible for retrieving images of diagnostic quality. With a high-speed hospital network, the high frame rate and lossless compression are not an issue anymore and there is no reason to limit the output frame rate of echocardiography systems. Aggressive lossy compression also isn't appropriate. Every echocardiography system suggests the option of lossless compression that is underused. Storing echocardiography studies isn't a formal process, but an option to retrieve anytime a high-quality image. An alternative is physician-controlled examinations directly on echo systems. Even in this case, there is a problem in case of revision of the diagnosis or future comparison with this exam.

Even when echocardiography examinations were transmitted with original frame rate and lossless compression was used, if the echocardiographic video loops are observed on a monitor outside echo system with a fixed refresh rate the interpretation and viewing of the study can be problematic.

According to studies conducted in radiology, eye strain occurs when the oculomotor systems work to maintain accommodation, convergence, and direction of gaze, resulting in symptoms like blurred vision, headaches, and eye pain (5). Digital displays may increase strain on the oculomotor system, overworking the eyes and resulting in eye strain, known as asthenopia (5) in addition to all the above-mentioned effects caused by a fixed non-adaptive refresh rate of the monitor. Image quality may contribute to errors in medical imaging perception (5) and it is known that suboptimal working conditions like stress, fatigue, and frustrations in the workplace may result in errors in medical imaging interpretation (6). In echocardiography, monitors should be optimized for the observation of echocardiography video clips, meaning that the refresh rate of the monitor should match the frame rate of the displayed video. Therefore, monitors with an adaptive refresh rate (that adapt their own refresh rate to the frame rate of the displayed video), are the best choice for echocardiography (2, 7). These are today available on the market.

## Conclusion

We suggest that acquisition frame rates should be maximalized, that clips should be stored at their original frame rates using lossless compression algorithms, and that echocardiography systems and view-stations should be tailored to high fidelity display of the acquired studies, especially, the use of arbitrary adaptive refresh rates of the monitors.

We conclude that technology has evolved considerably since the previous guidelines on digital storage and acquisition have been published. In the new era, every effort should be made to provide the added value of increased interpretation accuracy, reduced interpretation errors, and reduced operator “wear and tear” which can be accomplished at marginal additional cost.

## Data Availability Statement

The original contributions presented in the study are included in the article/Supplementary Material, further inquiries can be directed to the corresponding author.

## Ethics Statement

This research didn't require formal ethical approval as no human subjects were involved in the study.

## Author Contributions

ML: design and writing. VT: data analysis, figures preparation, and intellectual input. RB: a critical review and intellectual input. All authors contributed to the article, reviewed the manuscript, and approved the submitted version.

## Conflict of Interest

The authors declare that the research was conducted in the absence of any commercial or financial relationships that could be construed as a potential conflict of interest.

## Publisher's Note

All claims expressed in this article are solely those of the authors and do not necessarily represent those of their affiliated organizations, or those of the publisher, the editors and the reviewers. Any product that may be evaluated in this article, or claim that may be made by its manufacturer, is not guaranteed or endorsed by the publisher.

## Supplementary Material

Supplementary Video 1 The frame rate of the video and refresh rate of the monitor is the same. Background video, the apical 4-chamber view has a frame rate of 60 fps, vertical red lines move with a constant speed from frame to frame, in this case, 60 fps. There is an “agreement” between our eyes, red lines, and 4-chamber view. This video is easy for observation and perception.

Supplementary Video 2 The frame rate of the video is lower than the refresh rate of the monitor. Background video, the apical 4-chamber view has a frame rate of 45 fps, and vertical red lines move with a constant speed, simulating the refresh rate of 60 Hz. This video is difficult for perception at 60 Hz monitor, due to the significant jitter effect.

Supplementary Video 3 The frame rate of the video is higher than the refresh rate of the monitor. Background video, the apical 4-chamber view has a frame rate of 90 fps, vertical red lines move with a constant speed (refresh rate of the monitor is 60 Hz). This video is easier for perception than video 3 at 60 Hz monitor, but we also can't observe it for a long time due to the jitter effect and drop of frames.

## References

[1] Thomas JD Adams DB Devries S Ehler D Greenberg N Garcia M. Digital Echocardiography Committee of the American Society of Echocardiography. Guidelines and recommendations for digital echocardiography. J Am Soc Echocardiogr. (2005) 18:287–97. 10.1016/j.echo.2005.01.010 15746725

[2] Tyomkin V Vered Z Mor M Blondheim DS Carasso S Shimoni S. Is It Time to Revise the Guidelines and Recommendations for Digital Echocardiography? J Am Soc Echocardiogr. (2018) 31:634–6. 10.1016/j.echo.2018.01.021 29573930

[3] Davis J Hsieh YH Lee HC. Humans perceive flicker artifacts at 500 Hz. Sci Rep. (2015) 5:7861. 10.1038/srep07861 25644611 PMC4314649

[4] Watson AB. High Frame Rates and Human Vision: A View Through the Window of Visibility. SMPTE Motion Imaging J. (2013) 122:18–32. 10.5594/j18266XY

[5] Krupinski EA. Current perspectives in medical image perception. Atten Percept Psychophys. (2010) 72:1205–17. 10.3758/APP.72.5.1205 20601701 PMC3881280

[6] Sabih DE Sabih A Sabih Q Khan AN. Image perception and interpretation of abnormalities; can we believe our eyes? Can we do something about it? Insights Imaging. (2011) 2:47–55. 10.1007/s13244-010-0048-1 22347933 PMC3259345

[7] Thomas J. Reply to "Is It Time to Revise the Guidelines and Recommendations for Digital Echocardiography?” *J Am Soc Echocardiogr*. (2018) 31:636–8. 10.1016/j.echo.2018.02.002 29606332

